# Effects of Increasing Neuromuscular Electrical Stimulation Current Intensity on Cortical Sensorimotor Network Activation: A Time Domain fNIRS Study

**DOI:** 10.1371/journal.pone.0131951

**Published:** 2015-07-09

**Authors:** Makii Muthalib, Rebecca Re, Lucia Zucchelli, Stephane Perrey, Davide Contini, Matteo Caffini, Lorenzo Spinelli, Graham Kerr, Valentina Quaresima, Marco Ferrari, Alessandro Torricelli

**Affiliations:** 1 Movement To Health (M2H), EuroMov, University of Montpellier, Montpellier, France; 2 Movement Neuroscience, IHBI, Queensland University of Technology, Brisbane, Australia; 3 Dipartimento di Fisica, Politecnico di Milano, Milan, Italy; 4 Dipartimento di Elettronica, Informazione e Bioingegneria, Politecnico di Milano, Milan, Italy; 5 Istituto di Fotonica e Nanotecnologie, CNR, Politecnico di Milano, Milan, Italy; 6 Dipartimento di Medicina Clinica, Sanità Pubblica, Scienze della Vita e dell'Ambiente, Università degli Studi dell'Aquila, L’Aquila, Italy; Duke University, UNITED STATES

## Abstract

Neuroimaging studies have shown neuromuscular electrical stimulation (NMES)-evoked movements activate regions of the cortical sensorimotor network, including the primary sensorimotor cortex (SMC), premotor cortex (PMC), supplementary motor area (SMA), and secondary somatosensory area (S2), as well as regions of the prefrontal cortex (PFC) known to be involved in pain processing. The aim of this study, on nine healthy subjects, was to compare the cortical network activation profile and pain ratings during NMES of the right forearm wrist extensor muscles at increasing current intensities up to and slightly over the individual maximal tolerated intensity (MTI), and with reference to voluntary (VOL) wrist extension movements. By exploiting the capability of the multi-channel time domain functional near-infrared spectroscopy technique to relate depth information to the photon time-of-flight, the cortical and superficial oxygenated (O_2_Hb) and deoxygenated (HHb) hemoglobin concentrations were estimated. The O_2_Hb and HHb maps obtained using the General Linear Model (NIRS-SPM) analysis method, showed that the VOL and NMES-evoked movements significantly increased activation (i.e., increase in O_2_Hb and corresponding decrease in HHb) in the cortical layer of the contralateral sensorimotor network (SMC, PMC/SMA, and S2). However, the level and area of contralateral sensorimotor network (including PFC) activation was significantly greater for NMES than VOL. Furthermore, there was greater bilateral sensorimotor network activation with the high NMES current intensities which corresponded with increased pain ratings. In conclusion, our findings suggest that greater bilateral sensorimotor network activation profile with high NMES current intensities could be in part attributable to increased attentional/pain processing and to increased bilateral sensorimotor integration in these cortical regions.

## Introduction

Neuromuscular electrical stimulation (NMES) has been clinically demonstrated to improve movements of neurological populations with motor disabilities, such as post-stroke and incomplete spinal cord injury [1]. The principle of NMES is to apply repeated electrical currents to the peripheral motoneuronal axonal branches overlying the muscle of interest at a stimulation intensity that elicits muscle contractions. This in turn leads to the well characterised peripheral neuromuscular adaptations such as increased muscle strength and oxidative capacity [2–5]. More recently, it has been reported that rapid supraspinal central nervous system (CNS) reorganization/neuroplastic mechanisms are implicated during NMES [6–8]. This is because NMES activates peripheral sensory neuronal axons that send proprioceptive (and pain) afferent signals from the stimulated muscle to the CNS leading to cortical neuroplasticity and improvements in voluntary activation [6, 7] through a process of sensorimotor integration [7]. In addition, NMES current intensity above motor threshold is an important factor in determining the rapid cortical neuroplastic changes. In particular, NMES current intensities above motor threshold increases corticospinal excitability while NMES current intensities at sensory threshold decreases corticospinal excitability [7, 9].

Cortical correlates of NMES-evoked movements have been demonstrated in a few functional magnetic resonance imaging (fMRI) studies [10–14]. For instance, Blickenstorfer et al. (2009) observed a cortical activation pattern including the contralateral primary motor (M1) and sensory (S1) cortex (i.e., sensorimotor cortex-SMC) and bilateral secondary somatosensory area (S2), supplementary motor area (SMA), premotor cortex (PMC), and regions of the prefrontal cortex (PFC) during NMES-evoked wrist extension/flexion movements at current intensities just above the individual motor threshold (9–23mA) but minimising pain discomfort. Smith et al. [12] observed a positive relationship between increases in sensorimotor network activation and increases in NMES current intensity up to the motor threshold. Since NMES current intensities well above the motor threshold and up to the maximum tolerated current intensity (MTI) have been commonly utilized in strength and clinical neurorehabilitation programmes [5, 6], neuroimaging studies are necessary to investigate the cortical correlates of increasing NMES current intensity at the MTI on healthy subjects and patients.

A neuroimaging modality applicable in these studies is represented by functional near-infrared spectroscopy (fNIRS). This technique, exploiting the hemoglobin absorption spectra in the NIR range, gives a non-invasive measure of the hemodynamic changes in tissues such as the cortical microcirculation blood vessels (for review see [15, 16]). If compared with the other neuroimaging methods, fNIRS represents an optimal brain imaging monitoring tool given that it does not require stringent physical and motor constraints compared to fMRI. The principle of fNIRS lies in the mechanism of neurovascular coupling [17]. Cerebral blood flow adequate for brain activity and metabolic demand is maintained through feedforward and feedback processes of autoregulation and neurovascular coupling [17]. Thus when a specific brain region is activated, cerebral blood flow increases in a temporally and spatially coordinated manner tightly linked to changes in neural activity through a complex sequence of coordinated events involving neurons, glia, arteries/arterioles, and signalling molecules [17]. Therefore, fNIRS infers changes in neural activity that is mirrored by changes in blood oxygenation in the region of the activated cortical area (i.e., increase in O_2_Hb and decrease in HHb) [18].

Multichannel fNIRS neuroimaging has shown unilateral hand motor tasks activate regions of the cortical sensorimotor network, including the contralateral SMC, PMC/SMA, and PFC [19], which is consistent with fMRI neuroimaging [20–22]. Multichannel fNIRS neuroimaging has also shown an increase in activation of SMC and S2 regions during median nerve electrical stimulation just below the motor threshold current intensity (3–10mA) but minimising eliciting painful sensations [23, 24]. To the best of our knowledge, only our previous fNIRS study [25] has investigated the contralateral PFC activation induced by NMES of the elbow flexors (biceps bachii and brachioradials muscles) at increasing current intensities from well above motor threshold (45–55mA), which evoked strong muscle contractions with some pain perception, to MTI levels (55–70mA). That study found a relationship between the NMES current intensity to the MTI levels and the levels of PFC activation, which was most likely due to increased attentional demands and pain processing. However, the effects of increasing NMES current intensity to MTI levels on activation of the cortical sensorimotor network and perceived pain levels associated to the evoked-sensory/systemic physiological responses are still unknown.

In fNIRS neuroimaging, before reaching the deeper cortical region and exiting from the head, NIR photons must travel through the overlaying superficial layers (i.e. skin, subcutaneous tissue, aponeurosis, connective tissue, periosteum, cranium, meninges, cerebro-spinal fluid) [26]. Thus, the measured O_2_Hb and HHb concentration changes are a mixture of hemodynamic responses occurring in the cortical region and interfering responses occurring in these overlaying superficial (extra-cerebral) layers [27]. Recent reports have raised a question against the assumption that O_2_Hb/HHb changes measured by continuous wave (CW) fNIRS actually originated only from the cortical hemodynamic response (for review see [27–29]). Although several methods have been proposed to separate cortical and extra-cranial components in CW fNIRS signals (for review see [28, 30]), no consensus has been reached yet on the best approach. Since blood flow increase in the scalp can be expected during NMES we used the time domain (TD) fNIRS technique to exploit its ability in separating the superficial from the cortical layer fNIRS signals (for review see [31]). The ability of TD fNIRS to discriminate hemodynamic signals from intra- and extra-cerebral (i.e. cortical and superficial) layers has been already demonstrated through numerical simulations, experiments on tissue phantoms, and in vivo measurements [28, 32–41].

Although there have been several neuroimaging studies investigating neural activity accompanying NMES-evoked movements, these previous studies adopted low electrical stimulation not exceeding motor threshold, and hence the effects of high NMES intensities on neural activity, that is used practically in clinical neurorehabilitation programs, remains to be clarified. Such study will contribute to identify more effective neurorehabilitation programs. Therefore, the aim of the present study was to utilise a multi-channel TD fNIRS system to map the bilateral cortical sensorimotor network activation profile with increasing NMES current intensities from above motor threshold to the MTI, and with reference to the activation profile during VOL wrist extension movements. Considering the SMC, PMC/SMA, S2 involvement in sensorimotor integration, we hypothesized that greater cortical sensorimotor network (SMC, PMC, SMA, S2) activation would be induced with increased NMES current intensities; and considering the PFC involvement in attention and pain processing, we further hypothesized that the PFC would also show greater pain related activation at NMES current intensities at MTI levels.

## Materials and Methods

Nine male healthy volunteers (age: 39.2±13.0 y, height: 179.1±7.4 cm, weight: 81.7±15.7 kg) participated in this study. All subjects had no known health problems, no history of neurological disorders, and no upper extremity muscle or joint injuries. All subjects were right handed according to the Edinburgh handedness inventory [42].

### Ethics Statement

The study was in accordance with the Declaration of Helsinki and was approved by the Institutional Review Board at the Department of Physics, Politecnico di Milano. Written informed consent was obtained from all subjects.

### Experimental procedure

The study was conducted in a quiet and dimly lit room. Subjects were seated in a comfortable chair with both arms on a table. The right wrist extensor muscles (i.e., extensor carpi radialis longus, extensor carpi radialis brevis, extensor digitorum communi, and extensor carpi ulnaris) were stimulated (biphasic symmetrical rectangular pulse shapes at 30 Hz and 200 μs pulse width) with a pair of 5x5 cm self-adhesive electrodes (CEFAR Physio 5, DJO France SAS, Mouguerre, France). One electrode was located on the motor point of the right wrist extensor muscles, the other one on the distal end of the muscle near the wrist. Each subject’s MTI was first determined by using a series of 6–8 brief (3–5 s) electrically stimulated contractions with increasing current intensity. After each increase in current intensity, the participants were asked to report their tolerance to further increases in current intensity. Then, MTI was defined as the intensity of stimulation received when the subjects were unable to tolerate an increase in current intensity. Three attempts were made at an individual level in order to ensure a consistent MTI determination and the group-range of MTIs was between 26–50 mA with a group-average MTI of 35.4±6.8 mA. The fNIRS experimental session started at least 10 minutes after determining the individual MTI.

Each subject was asked to perform 10 blocks of voluntary (VOL) contractions, each consisting in a series of ten 70° wrist extensions (visually monitored by the experimenter) 1-s duration interleaved with 1-s rest (0° wrist extension). Consecutive blocks were separated by a 20-s interval. Five min after the end of the VOL contractions, the subjects underwent NMES at four increasing levels of current intensity based on their individual MTI: 10%MTI, 50%MTI, MTI, and above MTI (MTI+). The NMES conditions consisted of 10 blocks of 10 stimulations (1-s stimulation, 1-s rest) with a 20-s interval between blocks. A 2-min rest period separated each of the 4 NMES conditions. For the MTI+ condition, the first block was performed at the MTI current level determined prior to the start of the experiment. During each of the subsequent 9 blocks, each subject was asked after the 5^th^ stimulation if a further current increase could be tolerated. In the case of an affirmative response, the current was increased by 1 mA increments until the subject indicated their new tolerance level.

### Pain/discomfort ratings

At the end of each experimental condition subjective pain was measured using a pain rating scale (PRS) [43] and discomfort ratings using a visual analogue scale (VAS) [44]. Participants were asked to verbally rate the level of pain on the PRS from 0 (no pain) to 12 (extremely painful), and to manually mark the level of discomfort on the VAS, consisting of a 10-cm line with”no discomfort” on one end and”intolerable sensation” on the other end.

### Physiological measurements

During the experiment, physiological and autonomic parameters such as heart rate (HR), respiratory rate (RR) and skin conductance (SC) were continuously monitored at a 256 Hz sampling rate with a commercial physiological sensing system (FlexComp Infiniti System T7555M, Thought Technology Ltd., Montreal, Canada). The electrocardiogram electrodes for HR measurements were positioned with a standard configuration with the negative electrode placed on the right shoulder, the positive electrode placed on the lower center or left side of the chest (xiphoid process) and the ground electrode on the left shoulder. A respiration band for RR measurements was placed over the subject’s clothing at the mid chest level. SC was measured between the middle and the ring fingers of the left hand. Subjects were asked to keep their hand as still as possible to minimize artefacts in the SC readings. To observe variations of physiological parameters across conditions, HR, RR and SC measurements were averaged at an individual level within each experimental block.

### TD fNIRS instrumentation

**Fig 1:**
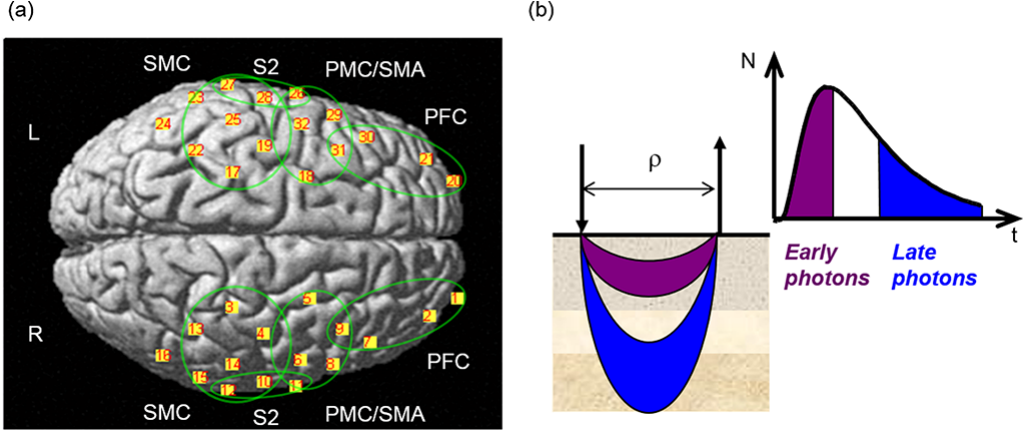
(a) Positions of the 32 fNIRS channels projected over a three-dimensional reconstruction of the brain cortex (L: Left hemisphere, R: Right hemisphere). The main cortical regions of interest are shown in the green circles: SMC- sensorimotor cortex (S1/M1); PMC/SMA- premotor cortex and supplementary motor area; PFC- prefrontal cortex; S2- secondary somatosensory area (See Table 1 for specific Brodmann areas representing each cortical region of interest). (b) Scheme illustrating the concept of early and late photons in TD fNIRS measurements (N is the number of photons, t the photon time-of-flight, and ρ the source detector distance).

The fNIRS measurements were performed by using a multi-channel dual wavelength TD fNIRS system developed at the Department of Physics, Politecnico di Milano, Italy [45, 46]. Two pulsed diode lasers (operating at 690 and 829 nm, with 80 MHz repetition rate) were used as the NIR light source. The NIR light pulses were time multiplexed over 10 locations, using optical fiber switches, each delivering an average power of ~0.2 mW. Diffused light was collected at 16 locations using single photon counting detectors based on multianode photomultipliers and processed by time-correlated single photon counting boards [47]. The fNIRS optodes were arranged symmetrically (5 sources and 8 detectors on each hemisphere) over the entire brain obtaining 32 measurements points (i.e. mid-point between source-detector pairs, see Fig 1A).

fNIRS optodes were positioned over the subjects scalp by means of a flexible electroencephalography (EEG) cap (g.EEGcap, g.tec medical engineering GmbH, Austria), presenting holes in correspondence to the positions of the International 10–20 system for EEG electrode placement (~3 cm source-detector distance). Home-made spring-loaded holders assured that optodes fitted into the holes and were firmly coupled to the skin. Moreover, the large holes on the cap allowed for good hair removal from under the optodes.

Since sufficient separation existed between optical channels in opposite hemispheres, a pair of light sources, one in each hemisphere, can be simultaneously activated without the risk of signal contamination. The sequential activation of the five pairs of sources every 0.2 s determined an overall acquisition time of 1 s for a full topographic map from 32 optical channels. Before the measurements, the count rate was set to ~50,000 ph/s for each wavelength which allowed for an acceptable value for the signal-to-noise-ratio during the measurements.

Cortical (intra-cerebral) and superficial (extra-cerebral) layer NIR light absorption changes, at experiment time *t* for wavelength λ, were calculated by means of the time-resolved Beer-Lambert formula in turbid media [48], eventually corrected to enhance the contribution from cortical (^intra^) layer and to remove possible disturbances caused by superficial (^extra^) layers [49]. The following formulas were used and a detailed description of the analysis can be found in [31]:

$$\Delta \mu_{a}^{extra}(t;\lambda )=-\ln[\frac{N(d_{E},w_{E};t;\lambda )}{N_{0}(d_{E},w_{E};\lambda )}]/L_{E}$$ (1)

$$\Delta \mu_{a}^{intra}(t;\lambda )=-\ln[\frac{N(d_{L},w_{L};t;\lambda )}{N_{0}(d_{L},w_{L};\lambda )}-\frac{N(d_{E},w_{E};t;\lambda )}{N_{0}(d_{E},w_{E};\lambda )}+1]/L_{L}$$ (2)

where N(*d*,*w*; *t*;λ) is the number of photons collected in a time window with delay *d* and width *w*, and N_0_(*d*,*w*;λ) is the number of photons, collected in a window with the same width, at the same delay and for the same wavelength, averaged over the resting period of the protocol. Parameters *d*_*E*_, *w*_*E*_ (*d*_*L*_, *w*_*L*_) represent delay and width of the time window corresponding to early (late) arriving photons, whereas *L*_*E*_ (*L*_*L*_) represent the pathlength of the early (late) photons (see Fig 1B). As in our previous studies [41, 50, 51], we have used *d*_*E*_ = 0 ps, *w*_*E*_ = 800 ps, *d*_*L*_ = 2,400 ps and *w*_*L*_ = 1,200 ps. A rough assumption for the path-length is *L* = *vτ*, where *v* is the speed of light in the medium and *τ* is the average photon time-of-flight. Therefore, in Eq.(1) and (2) we have used *L*_*E*_ = *vτ*_*E*_, and *L*_*L*_ = *vτ*_*L*_, with *τ*_E_ = 400 ps and *τ*_L_ = 3,000 ps calculated as average time-of-flight in the early and late time window, respectively. The number of received photons in the late time window were typically 6,000, with a coefficient of variation of 1.3% [45]. Making the assumption that hemoglobin is the only chromophore contributing to absorption, the time courses of O_2_Hb and HHb concentration changes were then derived by the Beer-Lambert law using the hemoglobin absorption spectra [27]. It should be noted that the thickness of the superficial layer was assumed to be constant across channels and subjects. However, choosing a time window of analysis with appropriate delay and width (e.g. *d*_*L*_ = 2,400 ps, *w*_*L*_ = 1200 ps) allows optimal representation of the photon migration through deeper (cortical) regions that accounts for inter-subject anatomical variability [52, 53].

### Statistical parametric mapping of TD-fNIRS signals

General Linear Model (GLM) analysis was applied to the time courses of O_2_Hb and HHb concentration changes (in superficial and cortical layers) by using NIRS-SPM software [54, 55] both at the single subject level and for the whole group. The positions of the 32 channels were registered over a reference MRI atlas in the Montreal Neurological Institute (MNI) coordinates system [56], and the points on the scalp were projected over a three-dimensional reconstruction of the brain cortex (see Fig 1A). Cortical regions corresponding to each of the 32 channels were extrapolated using the Anatomy 1.8 toolbox for SPM [57] (see Table 1).

**Table 1:** Cortical regions (Brodmann area, BA) corresponding to the 32 fNIRS channels on the left (LH) and right (RH) hemisphere (see Fig 1 for the location of individual channels). PFC: prefrontal cortex (BA 9, 10, 11, 46); SMC: primary sensorimotor cortex (S1: BA 1, 2, 3; M1: BA 4); PMC/SMA: premotor cortex and supplementary motor area (BA 6); S2: secondary somatosensory area (BA 43, 40); AG: angular gyrus (BA 39).

Cortical regions	LH Channel	RH Channel
PFC	20	1
PFC	21	2
PFC	30	7
PFC	31	9
PMC/SMA	29	8
PMC/SMA	18	5
PMC/SMA	32	6
S2, PMC/SMA	26	11
SMC, S2	28	10
SMC, PMC/SMA	19	4
SMC, PMC/SMA	17	3
SMC	25	14
SMC, S2	27	12
SMC	22	13
SMC	23	15
AG	24	16

Pre-processing algorithms were applied using internal routines of the NIRS-SPM software: a detrending filter (Wavelet-MDL) was used to remove physiological components from the NIRS signal due to breathing, cardiac, and vasomotion [54]. Time series (O_2_Hb or HHb time course) were modeled as a linear combination of *L* regressors (known functions) plus an error term ε for each of the *J* channels:

$$\underset{\left(T\times J\right)}{Y}=\underset{\left(T\times L\right)}{X}\underset{\left(L\times J\right)}{\beta }+\underset{\left(T\times J\right)}{\varepsilon }$$ (3)

where *X* is the design matrix (*T*x*L)* containing the L regressors (columns) defined over T time points (rows), and Beta is the matrix (LxJ) representing the weight parameter of each regressor fitting the original data by minimization of the least-squares error For each experiment, a design matrix for O_2_Hb was built by two regressors (*rest* and *task)* obtained through a convolution between the canonical hemodynamic response function (HRF, modeled as a linear combination of gamma functions) [58] and a step-function equal to 1 during the *task* periods, 0 elsewhere; the *rest* regressor was modelled with a step-function being 1 during the rest and 0 during the task, convolved with the HRF. Conversely, given that HHb is expected to decrease during the task, the two regressors for HHb were built as a convolution between the HRF and a step-function equal to -1 during the *task* periods, 0 elsewhere; the opposite step function was used for the *rest regressor*.

GLM analyses were performed on the single experimental condition (VOL and different NMES conditions) in order to investigate if task-related hemodynamic responses (e.g. changes of O_2_Hb and HHb signals) were significantly different from changes measured during resting conditions. For each condition, a T-test was conducted for each channel k on the expected value of ${\hat{\beta }}_{task}^{k}-{\hat{\beta }}_{rest}^{k}$. Furthermore the SPM method was used to compare the activations registered during different conditions. Thus a new design matrix was built composed only by the five *task* regressors for all the conditions. T-tests were performed on a new linear combination of coefficients, contrasting the regressors related to the *task* performed in different experiments. The different NMES conditions were compared firstly with the task period during the VOL condition (chosen as reference), and then between each of the NMES conditions, by following the hypothesis that O_2_Hb and HHb variations should increase when increasing the current intensity.

The T statistic was calculated for every channel, and sparse T-values associated to each optical channel were interpolated over the whole probe extension with a cubic interpolation. The p-values were finally inferred from the calculated T-values and graphically represented on a map in a logarithmic scale. The same visualization method of the statistical analyses has been previously used by Fazli et al. [59].

#### Statistical analysis of current intensity, pain/discomfort and physiological measurements

Statistical analyses were conducted with SigmaStat software (Systat Software Inc., Erkrath, Germany). All data were first examined for normality and homogeneity using Skewness-Kurtosis and Levene tests, respectively. A Friedman repeated measures ANOVA was used to determine whether current intensity amplitude level (mA) was different between the 4 NMES conditions (10%MTI, 50%MTI, MTI and MTI+). A one-way repeated measures ANOVA was used to determine whether each physiological measurement (HR, RR, and SC) and pain (PRS) rating was different across the experimental conditions (VOL, 10%MTI, 50%MTI, MTI and MTI+). A Friedman repeated measures ANOVA was used to determine whether discomfort (VAS) ratings were different between the experimental conditions (VOL, 10%MTI, 50%MTI, MTI and MTI+). When appropriate, the Tukey's HSD post-hoc test was used to find significant differences between pairs of conditions. Data are presented as mean±SD. The significance level was set at p<0.05.

## Results

### Current intensity, pain/discomfort ratings and physiological parameters

Table 2 shows the current intensities and pain/discomfort ratings for the experimental conditions. No subjective indications of pain or discomfort during the VOL condition were reported. For the 10%MTI condition, the current intensity was significantly lower compared to the other NMES conditions and below the motor threshold, such that wrist movements were not observed, and no or minimal pain/discomfort were reported by the subjects. As expected, at current intensities greater than the motor threshold (i.e. the 50%MTI, MTI and MTI+ NMES conditions) wrist extension movements were produced. The PRS (and VAS) scores were found to progressively increase from the 10%MTI (not painful/discomforting) to the 50%MTI (moderately painful/discomfort), and up to the MTI and MTI+ conditions (extremely painful, discomforting), which corresponded with the increase in current intensities (see Table 2). Although the VAS score and current intensity for MTI+ were significantly greater than the corresponding values at 50%MTI, they were both not significantly different from MTI.

**Table 2:** Grand average (n = 9, mean±SD) of the pain/discomfort and physiological parameters during the voluntary (VOL) and neuromuscular electrical stimulation (NMES) conditions at percentages of the individual maximal tolerated current intensity (MTI).

	Conditions				
	VOL	10%MTI	50%MTI	MTI	MTI+
**Current Intensity (mA)**	**-**	3.6±0.7* ^+^ ^#^	17.8±3.4* ^+^	35.4±6.8	64.0±14.2
**Pain/discomfort**					
** PRS** [0–12, a.u.]	-	0.2±0.3* ^+^ ^#^	1.6±1.0* ^+^	4.6±0.5*	6.9±1.2
** VAS** [0–10, a.u.]	-	0.9±0.3* ^+^ ^#^	4.1±0.7*	9.3±0.4	9.9±0.1
**Physiological parameters**					
** SC** [μS]	2.3±2.5* ^+^ ^#^	3.0±3.2*	3.5±3.6	3.8±3.7	4.1±3.9
** HR** [beats/min]	71.9±9.6	69.0±7.6* ^+^ ^#^ ^^^	71.2±7.8	71.7±8.3	73.7±9.1
** RR** [breaths/min]	15.4±0.7	14.9±0.8	15.0±0.8	14.8±0.9	14.6±0.7

PRS: pain rating scale; VAS: visual analogue scale; SC: skin conductance; HR: heart rate; RR: respiratory rate.

*: Significantly (*P*<0.05) different from MTI+

^+^: Significantly (*P*<0.05) different from MTI

^#^: Significantly (*P*<0.05) different from 50%MTI

^^^: Significantly (*P*<0.05) different from VOL.

The physiological parameters (SC, HR and RR) during the VOL and NMES conditions are shown in Table 2. Although SC during the NMES conditions with current intensity at 50%MTI and more (50%MTI, MTI and MTI+) was significantly greater than compared to VOL and 10%MTI, there were no significant differences between 50%MTI, MTI and MTI+. HR and RR remained relatively unchanged over the experimental conditions.

### Cortical and superficial O_2_Hb and HHb maps during VOL and NMES conditions

**Fig 2:**
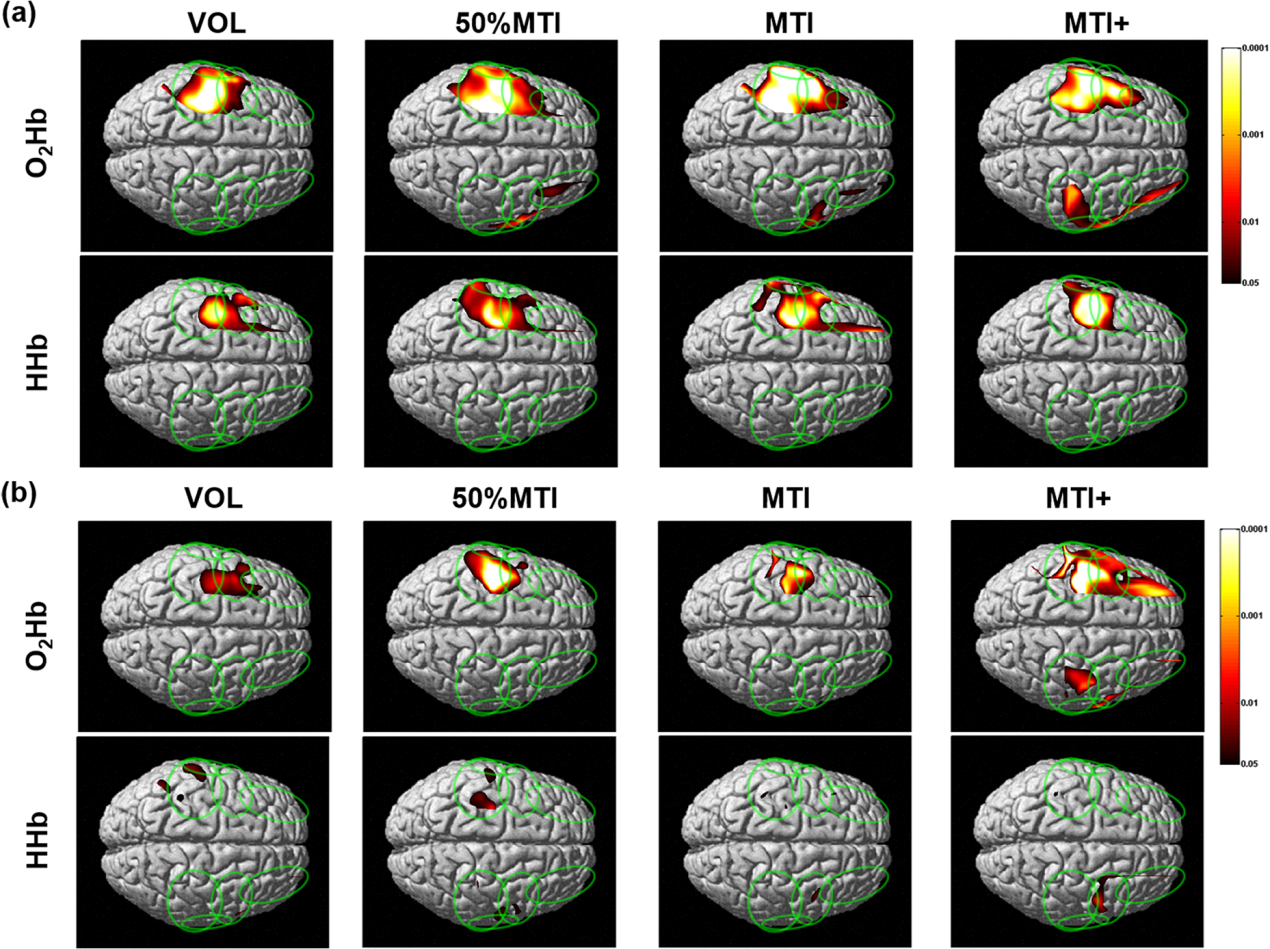
Group mean (n = 9) cortical (a) and superficial (b) oxygenated (O_2_Hb) and deoxygenated (HHb) hemoglobin maps during the voluntary (VOL) and NMES conditions at percentages of the individual maximal tolerated current intensity (MTI). The vertical colored scale indicates the significance level based on the uncorrected p-values (p<0.05-p<0.0001).

Fig 2 shows the O_2_Hb and HHb maps for the cortical and superficial layers during the VOL and NMES conditions. For the VOL condition, the cortical layer O_2_Hb and HHb maps showed significant task related activations (i.e., O_2_Hb increase and HHb decrease) in the contralateral (left) hemisphere, primarily in the SMC and PMC/SMA regions; while the ipsilateral hemisphere showed no significant O_2_Hb increases or HHb decreases (see Fig 2A). The superficial layer O_2_Hb maps also showed a task related activation in the sensorimotor network area of the contralateral hemisphere (see Fig 2B), but these areas were narrower and characterized by a lower statistical significance than the corresponding area in the cortical layer. This was even more pronounced in the superficial layer HHb maps, where very narrow and not co-located areas with lower statistical significance were found compared to the cortical layer.

The GLM analysis indicated no significant superficial or cortical layer O_2_Hb increases or HHb decreases for the 10%MTI condition (data not shown in Fig 2). The O_2_Hb and HHb maps both showed that the NMES conditions that evoked wrist extension movements (50%MTI, MTI and MTI+) induced a significant area of activation in the cortical layer of the contralateral (left) sensorimotor network (primarily SMC, PMC/SMA, and S2) and PFC regions, and a smaller area of significant O_2_Hb increase in the ipsilateral hemisphere (see Fig 2A). Although, the NMES conditions also induced significant O_2_Hb increases in the superficial layer of the contralateral sensorimotor network (see Fig 2B), these areas were narrower (in the case of 50%MTI and MTI) or more spread out (in the case of MTI+) than in the cortical layer. Similarly, the superficial layer HHb maps showed a minimal area of HHb decrease with low significance in the contralateral sensorimotor network regions for the NMES conditions.

### Comparison of cortical O_2_Hb and HHb maps between NMES and VOL conditions

**Fig 3:**
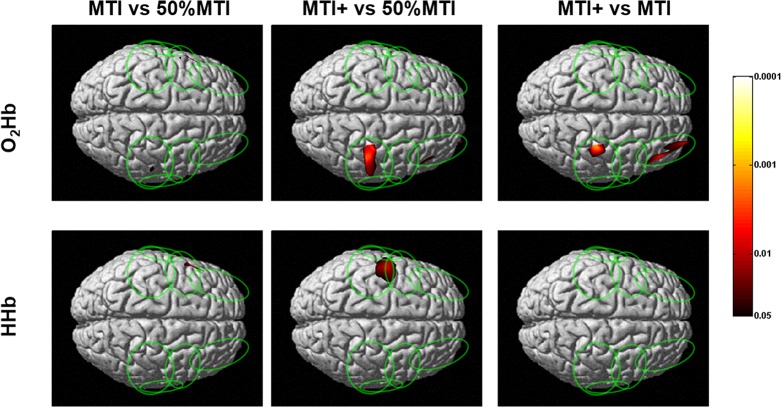
Group mean (n = 9) cortical oxygenated (O_2_Hb) and deoxygenated (HHb) hemoglobin contrast maps between the NMES conditions at percentages of the individual maximal tolerated current intensity (MTI). The vertical colored scale indicates the significance level based on the uncorrected p-values (p<0.05-p<0.0001).

The cortical layer O_2_Hb and HHb contrast maps (i.e. the representations of the statistical significance resulting from the comparison of different NMES conditions) between the three NMES conditions (50%MTI, MTI and MTI+) are shown in Fig 3. When contrasting MTI/MTI+ with 50%MTI, although the O_2_Hb maps showed no (MTI+) or minimal (MTI) greater activation of the contralateral sensorimotor network compared to 50%MTI, the HHb maps revealed greater activation in the contralateral sensorimotor network corresponding to the SMC and PMC/SMA regions. Furthermore, the O_2_Hb maps revealed a significantly greater area of activation in the ipsilateral sensorimotor network for both the MTI and MTI+ conditions compared to 50%MTI. For the contrast between MTI and MTI+, only the O_2_Hb maps showed a significantly greater area of activation in the ipsilateral sensorimotor network and PFC regions for the MTI+ than MTI condition, and no significant difference in the area of activation in the contralateral hemisphere.

**Fig 4:**
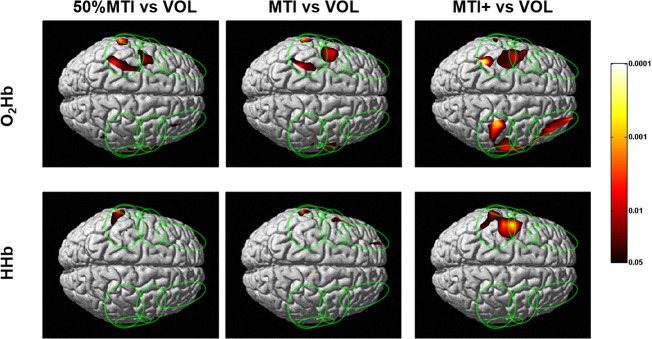
Group mean (n = 9) cortical oxygenated (O_2_Hb) and deoxygenated (HHb) hemoglobin contrast maps between the voluntary (VOL) and NMES conditions at percentages of the individual maximal tolerated current intensity (MTI). The vertical colored scale indicates the significance level based on the uncorrected p-values (p<0.05-p<0.0001).

The cortical layer O_2_Hb and HHb contrast maps between the VOL and NMES conditions are shown in Fig 4. The O_2_Hb and HHb contrast maps indicated that 50%MTI, MTI and MTI+ activated a significantly greater area of the contralateral sensorimotor network regions compared with the VOL condition (see Fig 4). Furthermore, the O_2_Hb maps indicated that compared with the VOL condition, the 50%MTI, MTI and MTI+ conditions significantly activated also the ipsilateral sensorimotor network regions, including the PFC regions for the MTI+ condition.

## Discussion

To the best of our knowledge, the present TD fNIRS study was the first to determine the cortical sensorimotor network activation profile and pain responses induced by NMES-evoked wrist extension movements at increasing current intensities relative to the individual maximal tolerated current intensity (MTI), and with reference to those obtained in response to voluntary (VOL) wrist extension movements. The cortical O_2_Hb and HHb maps indicated that NMES-evoked wrist extension movements (50%MTI, MTI, and MTI+) activated a contralateral sensorimotor network (SMC, PMC/SMA, S2) as that during VOL wrist extension movements, which is consistent with previous fMRI studies that compared between NMES-evoked and VOL movements [10, 13]. The novel findings were that i) when the NMES current intensity was increased from 50%MTI (mild pain/discomfort) to MTI/MTI+ (extremely painful/discomfortable), distinct patterns of bilateral sensorimotor network (including PFC) activation were evident (Figs 2A and 3); ii) compared to the VOL condition, NMES at high current intensities activated the contralateral sensorimotor network in a larger area and with higher significance, and activations were also seen in the ipsilateral sensorimotor and bilateral PFC regions (Figs 2A and 4).

### Cortical activation during NMES and VOL conditions

NMES-evoked movements are induced by applying to the skin overlying the muscles of interest repeated electrical currents at sufficient intensity to activate the peripheral motoneuronal axons that elicit the excitation-contraction coupling process for muscle force production. Although less well characterised, the NMES electrical field also activate peripheral sensory neuronal axons that send proprioceptive (and nociceptive) signals from the stimulated muscle to the CNS leading to supraspinal neural adaptations [5–7]. We have shown that unilateral NMES-evoked wrist extension movements (50%MTI, MTI and MTI+) activated primarily a contralateral sensorimotor network comprising the SMC, PMC/SMA and S2 regions, which are in agreement with previous fMRI findings of NMES-evoked movements [10, 11, 13, 60]

As expected, the unilateral VOL wrist extension movements were characterized by activation of contralateral sensorimotor network regions (SMC, PMC/SMA, S2) that are well known to be activated to perform hand motor tasks [19, 20, 22]. Furthermore, the regions of the contralateral sensorimotor network activated during VOL wrist extension movements were also activated during NMES-evoked wrist extension movements (Fig 2A). These findings are also in agreement with fMRI studies that compared the cortical sensorimotor network activation profile between VOL and NMES-evoked movements at current intensities at the motor threshold [10, 13]. A new finding was that NMES at painful/discomfortable current intensities (50%MTI, MTI, and MTI+) activated a greater and wider area of the contralateral sensorimotor network compared to VOL. It also activated regions of the ipsilateral sensorimotor network including bilateral PFC, which was particularly evident for the extremely painful/discomfortable NMES conditions (MTI and MTI+ conditions, Fig 3). It is possible to hypothesize that the greater bilateral sensorimotor network and PFC activation profile for the NMES conditions at high current intensities are related to greater pain processing in these cortical regions. These observations support our previous study [25] that showed an increased PFC activation with increasing NMES current intensities at MTI levels, and add to the previous studies that have alluded to the activation of the attention and pain processing network with NMES current intensities above the motor threshold [11, 14].

The pain processing network comprising the S1/S2 (pain intensity) and PFC (attention and evaluation of an emotional response to pain) regions receives parallel inputs from multiple nociceptive pathways, suggesting that pain is processed in a distributed fashion [61]. If a stimulus is intense enough to activate peripheral nociceptors, multiple brain areas of the pain matrix respond to this input in a correlated manner with perceived pain intensity [62, 63]. This schema is supported by the increased pain/discomfort scales, SC responses (a physiological measure of subjective pain/arousal [64]) and bilateral sensorimotor network activation profile during high NMES current intensities (Table 2 and Fig 2A). The S1, S2 and PFC are amongst those brain regions that consistently respond to painful/uncomfortable stimulations [61, 63, 65–70], and the PFC is densely interconnected with sensory areas [71], which integrates motor with sensory/proprioceptive information [72]. The M1 is also known to be implicated in pain processing [73]. The exercise physiology and clinical benefits of NMES have been proposed to be *via* a sensorimotor integration mechanism; increased proprioceptive signals from NMES-evoked movements activate the sensorimotor network, particularly the SMC, thereby increasing corticospinal excitability, and facilitating greater voluntary activation of the relevant neuronal network [6, 74]. Accordingly, it can be suggested that NMES-evoked wrist extension movements at high stimulation intensities activate brain regions related to sensorimotor integration, which provides evidence for the concept that NMES-evoked somatosensory and nociceptive inputs lead to changes in M1 excitability [6, 7], which in turn can cause functional improvements, including muscle activation and strength [6].

The detection of bilateral sensorimotor network activation elicited by unilateral NMES of the wrist extensors at high current intensities is consistent with the presence of transcallosal interactions between the two hemispheres [75]. These observations signify the interhemispheric integration of the somatosensory and nociceptive information primarily at the level of the SMC (but also PMC/SMA, S2 and PFC), and provide support for the concept of distributed (bilateral) processing in cortical sensorimotor networks during unilateral NMES of the wrist extensors as previously shown for NMES of the wrist [11] and finger [13] extensors/flexors, unilateral median [24, 76] and peroneal [14] nerve stimulation, and unilateral voluntary movements with increased task demands [77–79]. A scheme for the bilateral activation of cortical networks with unilateral VOL and NMES is proposed: During VOL wrist extension movements, the ipsilateral sensorimotor network processing of sensory and motor information was inhibited prior to and during movement execution through interhemispheric inhibitory processes [80] to allow the contralateral sensorimotor network to perform the right arm wrist extension movements, which was evidenced with robust activation of the contralateral sensorimotor network with no obvious ipsilateral activation. The ipsilateral inhibition may be aimed at reducing interference, aiding focus and thereby achieving high manual dexterity in unilateral voluntary motor tasks [81]. During NMES-evoked wrist extension movements, since it has been previously shown that unilateral median nerve stimulation at low current intensities activates contralateral sensorimotor regions while actively inhibiting ipsilateral sensorimotor regions [82], NMES at high stimulation currents may disinhibit ipsilateral sensorimotor network to process somatosensory and nociceptive information, which was evidenced in the present and a previous [14] study with bilateral activation of the sensorimotor network. Therefore we hypothesize that NMES at high current intensities is able to modify the excitability of interhemispheric connections and perhaps the balance between interhemispheric excitation and inhibition [6]. Further neurophysiological research is necessary to confirm this hypothesis.

### Cortical hemodynamic measurements using TD fNIRS

The O_2_Hb and HHb maps of the deeper layers (including mainly the cerebral cortex) revealed a greater area of activation (i.e. significant increase of O_2_Hb and a corresponding significant decrease of HHb) during the experimental conditions (Fig 2A) which was not found in the superficial layers (including mainly the scalp skin) where mainly O_2_Hb maps showed significant statistical effects (Fig 2B). This behavior of O_2_Hb is typical of a greater skin blood efflux probably due to increasing pain/discomfort sensation by the subjects as the NMES current intensity was increased. This emotional state of the subjects was confirmed by the subjective pain/discomfort scales and SC responses, which increased over the NMES current levels, while HR and RR remained at a similar level of that one observed in the VOL condition (Table 2). These findings suggest that attentional/pain/arousal processing in the sensorimotor network and PFC rather than systemic physiological mechanisms were primarily responsible for the changes in cortical O_2_Hb and HHb maps.

The superficial layer O_2_Hb maps also increased with increase of the stimulation current intensity in good spatial agreement with the cortical O_2_Hb maps. These findings confirm the results by Kirilina et al. [28] who demonstrated that the spatial agreement between cortical and superficial O_2_Hb changes are located in the superficial blood vessels supplying/draining the cortical layers. This suggests that the source of task-evoked superficial systemic signals in fNIRS (primarily O_2_Hb signals) is co-localized with veins draining the scalp. Conversely, the HHb maps of the superficial layer were found unchanged or minimal changes during all experimental conditions. The poor changes in HHb maps of the superficial layer compared with the well localized and significant HHb maps at the cortical layer confirm that the greater activation found in the cortical HHb maps arose primarily from the underlying cortex. Therefore, we are confident that the cortical activation maps found during NMES and VOL conditions arose from the cerebral cortex and were not an artefact of the extracerebral systemic haemodynamic changes.

## Conclusion

We have demonstrated that, compared to the VOL wrist extension movements, NMES induces a greater bilateral sensorimotor network activation profile with increasing stimulation intensities and pain ratings. This could partly be explained by the increased attentional/pain processing associated with higher stimulation current intensities and to the increased bilateral sensorimotor integration in these cortical regions. Future studies should apply TD fNIRS neuroimaging in longitudinal patient studies for assessing cortical neuroplastic changes associated with NMES neurorehabilitation treatment.
